# Development cooperation for health: reviewing a dynamic concept in a complex global aid environment

**DOI:** 10.1186/1744-8603-8-5

**Published:** 2012-03-15

**Authors:** Peter S Hill, Rebecca Dodd, Scott Brown, Just Haffeld

**Affiliations:** 1 Australian Centre for International and Tropical Health, The University Of Queensland, Herston Road Herston, 4006 Queensland, Australia; 2 AusAID Health Resource Facility, Canberra, Australia; 3 School of Population Health, The University Of Queensland, Herston Road, Herston 4006 Queensland, Australia; 4 University of Oslo, Medical Faculty, PO Box 1078, Blindern 0316 Oslo, Norway

**Keywords:** Coordination, Harmonisation, Alignment, Aid Effectiveness, Paris Principles, Paris Declaration, Accra Action Agenda, Sector-Wide Approaches

## Abstract

The 4^th ^High Level Forum on Aid Effectiveness, held in Busan, South Korea in November 2011 again promised an opportunity for a "new consensus on development cooperation" to emerge. This paper reviews the recent evolution of the concept of coordination for development assistance in health as the basis from which to understand current discourses. The paper reviews peer-reviewed scientific literature and relevant 'grey' literature, revisiting landmark publications and influential authors, examining the transitions in the conceptualisation of coordination, and the related changes in development assistance. Four distinct transitions in the understanding, orientation and application of coordination have been identified: coordination within the sector, involving geographical zoning, sub-sector specialisation, donor consortia, project co-financing, sector aid, harmonisation of procedures, ear-marked budgetary support, donor agency reform and inter-agency intelligence gathering; sector-wide coordination, expressed particularly through the Sector-Wide Approach; coordination across sectors at national level, expressed in the evolution of Poverty Strategy Reduction Papers and the national monitoring of the Millennium Development Goals; and, most recently, global-level coordination, embodied in the Paris Principles, and the emergence of agencies such as the International Health Partnerships Plus. The transitions are largely but not strictly chronological, and each draws on earlier elements, in ways that are redefined in the new context. With the increasing complexity of both the territory of global health and its governance, and increasing stakeholders and networks, current imaginings of coordination are again being challenged. The High Level Forum in Busan may have been successful in recognising a much more complex landscape for development than previously conceived, but the challenges to coordination remain.

## 

In late November 2011 global stakeholders met in Busan, South Korea for the 4^th ^High Level Forum on Aid Effectiveness, planning the next iteration in the evolution of aid effectiveness. The Aid Effectiveness Agenda has roots that extend well before the 2005 declaration of the Paris Principles, with their emphasis on ownership, alignment, harmonisation, managing for results and mutual accountability [1]. For health, the 1978 Alma-Ata declaration, which focussed international attention and resources on Primary Health Care, also marked a more fundamental paradigm shift-establishing the notion of international responsibility for health, the idea that rich countries should help poorer ones to achieve health goals [2].

With international responsibility came increasing formalisation of arrangements for development assistance and-in parallel with the growth of the broader aid industry-an increasing number of development partners active in the health sector. Within two decades there were complaints of an "unruly mélange" of development initiatives providing support to health [3]. Indeed, calls for better *coordination *of aid are almost as common as calls for more aid, and have remained a central theme of every subsequent international health initiative since Primary Health Care.

This review examines how the concept of aid coordination, and its application to health systems, has evolved over the last two decades, and provides a base from which to consider further change. In this time international health has been re-shaped by globalisation, confronted by transitions in health and disease and has coped with unanticipated and resurgent epidemics. The development landscape has also changed substantially, with new resources, new players and new relationships, in turn leading to new forms of global health governance [4]. In this dynamic and shifting environment one idea has retained traction: that aid needs to be better managed and co-ordinated. Though, as we describe below, the ways in which that idea has been packaged and articulated, and the parameters of who should be engaged and to what end, have all evolved considerably.

Our review draws on the peer-reviewed scientific literature and relevant 'grey' literature-reports, unpublished research and analyses, and web based materials. Initial searches used engines including PubMed, Medline Ovid, CINAHL and Google Scholar, using combinations of the search terms: 'aid coordination', 'development assistance', 'aid', 'cooperation', 'harmonisation', 'alignment', 'aid effectiveness', from 1990. Given the breadth of some of these terms, the initial searches yielded a vast quantity of relevant and irrelevant material, and in particular, access to grey literature has required a purposive strategy tracking those references most commonly cited. Inevitably, the analysis has revisited landmark publications and influential authors, showing how their work has become an integral part of the discourse on aid coordination (and later 'aid effectiveness'), both influencing donor practice and shaping our interpretation of it.

Our point of departure is the early 1990s, when the issue of coordination became a focus for international attention. From there we trace four distinct transitions: coordination within the sector; sector-wide coordination; coordination across sectors at national level; and, most recently, global-level coordination. There is an apparent progression in the levels at which coordination is sought, as the drivers for coordination have shifted from local integration of development initiatives, to the management of sectoral resources, to the more comprehensive goals of aid effectiveness, linked with the need to make order of a global aid landscape that has become increasingly complex. The transitions are largely but not strictly chronological--as each new conceptualisation of coordination is unpacked, it draws on earlier elements but in ways that are redefined in the new context [5].

## Coordination within the sector

Early attempts to improve donor coordination in the health sector emerged in response to growing evidence of duplication and fragmentation in donor support [6]. The project delivery format was itself identified as a problem, with its short time-lines, limited budget and high transaction costs. Cassels suggested that *"in many of the poorest countries there are arguably too many projects already, swamping limited administrative and managerial capacity with the needs of detailed project design and monitoring*"[7]. The result was an increasing administrative burden for recipient governments, which both stretched and undermined their authority [8].

The instruments of coordination were essentially a formalisation of attempts by donors to coordinate activities within the sector amongst themselves: geographical zoning, sub-sector specialisation, donor consortia, project co-financing, sector aid, harmonisation of procedures, ear-marked budgetary support, donor agency reform and inter-agency intelligence gathering [3,9].

One of the first serious critiques of coordination was offered by Buse and Walt, observing that the "*concept of coordination has been embraced by the health policy community but has remained ill-defined*", and indicating that the term was used interchangeably with "*coherence, compatibility, cooperation, collaboration, consultation, concertation, integration, harmonization, synchronization and even control and discipline*" [8]. None of these definitions, they argued, specified who coordinated whom or what, and to what purpose. Further, coordination was often donor-driven and inputs-focussed, and failed to connect with government counterparts-indeed, donor efforts were sometimes set up in parallel to government's own attempts at coordination [10].

In addition to critiquing current practise, Buse and Walt offered a way forward, suggesting that coordination should be defined as: "any activity or set of activities, formal or non-formal, at any level, undertaken by the recipient in conjunction with donors, individually or collectively, which ensures that foreign inputs to the health sector enable the health system to function more effectively, and in accordance with local priorities, over time" [8].

This emphasis on government control of the coordination process, along with the shift from *ad hoc *coordination in specific technical areas to a more comprehensive approach, were both hallmarks of the next iteration of coordination: the sector-wide approach.

### Sector-wide coordination

Sectoral Investment Programmes (SIPs), introduced in the late 1990s by the World Bank, appeared to offer a solution to this fragmentation of development assistance and the patchwork approach to coordination, with their focus on integration and the development of coherent programmes, in preference to individual projects [11]. More radical still, consistent with a desire to strengthen government control of its external resources, the approach aimed to engage all donors active in the sector and commit them to common implementation and monitoring arrangements [12,13].

SIPs contained the key elements of what would later be called the Sector-Wide Approach, or SWAp (the key difference being that SIPs were closely associated with the World Bank, while SWAps were not). Firstly, coordination would no longer rest in the hands of the donors; government would be "in the driver's seat":

"Here is my program in this sector: if you wish to help me implement it, you are most welcome. If you wish to do something different, I regret that you are not welcome in this sector in this country" [12].

Secondly, the level and scope of coordination would be ramped up: the SWAp would embrace the whole of the health sector within its parameters of coordination. Third, dialogue between donors and government would move upstream, from discussion of project implementation arrangements to the construction of national policy frameworks. Implicit in this last shift was a greater focus on the health systems that underpin disease programmes. In return for this influence donors would pool their finances and commit to implementation of the comprehensive package of reforms.

SWAps represented a paradigmatic shift: from coordination practice *within *the sector to an aspirational management *of *the sector and its resources--domestic and external--linked with an increasing focus on health systems strengthening by both the World Bank and the World Health Organization (WHO) [9]. Yet, as with intra-sectoral coordination, ambitious theorising was swiftly followed by a more hard-headed critique of the difficulties of implementing SWAps in practice. The tensions implicit in donor-partner asymmetry were immediately evident. "Complex coordination mechanisms" observed Pavignani and Hauck from the Mozambique SWAp, "have in some cases meant that additional TA [technical assistance] is required to address the lack of local capacity, which is at odds with the original objective of ensuring that recipients are in control" [14]. Similarly, it was argued that strong donor input could result in external partners dominating the policy process, with the result that policies reflected donor priorities but had little local support [15].

In addition, while many bilateral donors were supportive of SWAp concepts, they were constrained by their own administrative rules from participating in arrangements like pooled funding or the use of national systems for procurement or monitoring. Those who did adopt such systems risked problems associated with weak public administration and poor fiduciary accountability-issues that are beyond the capacity and jurisdiction of the health sector to influence [16]. Thus, use of government systems and procedures quickly segued to an emphasis on intent-or 'progressive reliance on' government systems-rather than full adoption of them [17].

As a result, the comprehensive vision of the SWAp had to be revised. Elements were implemented selectively, depending on needs and circumstances [18-20]. Few--if any--SWAps engaged all donors or all parts of the sector. Pooling of finances became the exception rather than the rule, with projects remaining the dominant mode of aid delivery, despite professed support by donors for more programmatic approaches [9,10,21].

### Coordination at national level

While the revolutionary promise of SWAps was not fully realised, the persuasiveness of its central arguments for local leadership, alignment with local policy, and harmonisation of procedures have endured in national level coordination mechanisms [1]. Their influence was also evident as the Development Assistance Committee of the Organization of Economic Cooperation and Development progressively sought to shape the contribution of its members, the G20, towards more effective development assistance, ultimately documented in the 2005 Paris Declaration on Aid Effectiveness [22].

Indeed, the challenges encountered by SWAps in using government systems and influencing policy development processes persuaded some donors of the need to move their policy focus even further upstream, laying the foundations for a whole of government approach. The concept of pooled financial resources-the least successful of any of the SWAp elements in practice-re-emerged in the form of channelling resources either 'on-budget' or 'through budget', which were also central to the next evolution of coordination efforts: Poverty Reduction Strategy Papers (PRSPs). Originally a World Bank requirement, devised to ensure that countries qualifying for debt relief spent newly-freed-up resources on poverty reduction programmes, PRSPs quickly became a means of coordinating government poverty reduction initiatives in different sectors and also for driving harmonization and alignment of donor support [23].

When the link with debt relief ended, PRSPs became simply poverty reduction strategies, the generic term indicating that each country had their own name, timetable and approach for this process. The key principles remained the same: policy development should be led by government; and aid should be channelled through government systems both to strengthen those systems and to ensure aid dollars were spent exclusively on government priorities [7,24].

As with SWAps, critiques of the Poverty Reduction Strategy Papers emerged early. Top among them were that PRSPs followed a generic blueprint provided by their main donor [25], that they prioritised economic growth over sectoral priorities, and that they failed to allocate additional resources to the social sectors [26,27]. And-as with SWAps-that while donors benefitted by rapid disbursement and reduced administrative costs, weak government capacity to implement made it impossible to ensure results [28]. Equally, stories of poor fiduciary and accountability systems fuelled familiar stories of corruption and wasted aid dollars.

### Towards global coordination

These critiques, alongside the increased attention to poverty and development associated with the Millennium Declaration and Millennium Development Goals (MDGs), prompted the growth of new aid initiatives which has characterised the latest phase of 'global' (as opposed to international) health [2]. The 2000s were marked not only by substantially increased funding for health, but also by a greater diversity of stakeholders and a multiplication of global health initiatives-with global civil society networks and private foundations rivalling traditional development agencies in terms of both resources and influence. The health aid architecture of the previous century, with the World Bank competing with WHO for leadership had now evolved into more diffuse governance structures, with no single authority dominating [29-31].

The consequences for coordination efforts have been profound, with new, complex inter-relationships emerging between different types of development partners-whose mandates range from the extremely narrow (a single disease) to the very broad (poverty reduction). In addition, even as the development landscape becomes more diverse so there are new global pressures to ensure coordination mechanisms at *every *level, from national to sectoral to disease specific. This has in turn spawned an increase and diversification of coordination mechanisms themselves, and a further re-packaging of the coordination concept in search for renewed authority.

At the top of the new, global coordination hierarchy is the series of high-level forums on aid effectiveness (Rome, Paris, Accra and Busan), through which development agencies and recipient countries, as well as global health initiatives such as the GAVI Alliance (GAVI) and the Global Fund to fight AIDS, Tuberculosis and Malaria (Global Fund), commit to measurable targets on alignment and harmonisation. As with SWAps, however, there is criticism that the emphasis has been on "intent rather than achievement" [17] with high levels of in-principle commitment but "highly uneven" implementation [32]. Donors have been accused of an "unfortunate mix of risk avoidance and 'political correctness'" avoiding full alignment through "transitional mechanisms" that essentially maintain the status quo, persisting with conditionality that ensures their preferred outcomes, but hesitating to confront corruption, ineffectual systems and project perks in partners [33]. But not all analyses are negative: a recent review by Dickenson argues that, by focussing on national systems and processes, the aid coordination agenda is creating the conditions for sustained improvements in health outcomes [34].

Whatever the differences of view on its impact, the Aid Effectiveness Agenda retains a central position in current debates on development assistance, and is in effect the latest, most dominant re-framing of the discourse on coordination. The 2007 International Health Partnership and Related Initiatives (IHP+) represents the health sector's response to this reframing. IHP + links global and national coordination processes by using global-level meetings (and advocacy) to promote investment in a single national health plan and monitoring framework, as well as other targets for better coordination of health aid. Global and national "compacts" commit recipients and donors to these targets, and provide a mechanism for monitoring. Like SWAps, IHP + compacts seek to create a substantive dialogue on policy issues, but in line with the continual re-formulation of approaches to coordination, have created a new mechanism for doing so: the Joint Assessment of National Strategies (JANS) [35]. The partnerships within IHP + have enabled both the World Bank and WHO access to new global funds, and new collaborations for achieving their health systems goals, with GAVI and the Global Fund (both signatories of the IHP global compact) proposing the JANS process for funding applications in the collaborative Health Systems Funding Platform [36].

### A complex systems perspective

The past decade has seen increasing complexity, both in terms of development assistance for health and its governance. The donor community itself has changed: the 20^th ^century dominance of the multi-lateral agencies and key bilateral agencies has been challenged by emerging actors from philanthropy and the private sector. But it is not simply the increasing numbers of development actors that typifies aid as a Complex Adaptive System, it is their heterogeneity, and their rich interactions through new--and dynamic--networks and alliances [4], in ways that have challenged global governance, raising questions of global accountability [2]. There has also been a corresponding--arguably consequent--burgeoning diversification of national government agencies and civil society organisations to respond to the raft of new funding and programmatic opportunities [37]. Severino and Ray argue that the current, dominant mechanisms of coordination of aid--the Creditor Reporting System of the Organisation for Economic Cooperation and Development (OECD), the monitoring of the Aid Effectiveness Agenda, the progress towards the MDGs--are out of step with this diversification, and no longer capture the full scope of development activity [37]. New donors from outside the OECD such as the BRICS--Brazil, Russia, India, China, South Africa--and philanthropies, foundations and the private sector are increasingly active providers of development assistance. Yet many do not feel bound by the norms and agreements of 'good donorship'. Thus conventional representations of the "donor community" may no longer be accurate. There are more actors, but it is their heterogeneity that compounds the complexity of global governance: their "different legitimacies, motivations, understandings, assumptions and discourses coexist, interact and often oppose one another"[38].

Two and half decades on, has the "unruly mélange" [3] triumphed? The latest re-packaging of the coordination agenda in the health sector suggests not. Indeed, health may be ahead of the curve with its response to the latest evolution in the aid architecture. First, there is a dedicated effort in the health sector to bring new foundations and partnerships into coordination processes, through efforts such as IHP+. Second, and partly as a result of this new configuration of stakeholders, the long-standing debate on targeted "vertical" programs versus the "horizontal" health systems strengthening is now being played out in the aid effectiveness arena, with so-called "diagonal" approaches [39] guiding alignment and harmonisation of donor activities at country level [40]. This roots coordination efforts in a substantive policy agenda, and helps counter criticism that aid effectiveness is too process oriented.

At the same time, though, doubts about the value of coordination efforts remain a persistent part of the aid discourse. Echoing earlier critiques of SWAps, Severino and Ray argue that "*more *coordination and harmonization is not always *better*", pointing to the costs of donor coordination, and the need for an optimal balance that allows for "better coherence while leaving space for a healthy level of diversity and emulation" [38]. Current coordination mechanisms themselves need to be seen as policy players in their own right, "competing with other policy communities for influence, resources and institutional space in the governance of health policy" [41].

The complexity of both the territory of global health, and its governance, does not suggest that a universal consensus is imminent. Understanding both as complex adaptive systems [4] makes sense of the dynamic and changing relationships between partners, and the constant reconfiguring of the elements of global health coordination [5]. But while the recent preparations for Busan suggest a more nuanced understanding of the dynamic development landscape, the original drivers of coordination--the desire for local management of a more cohesive and effective collective aid presence--risks being lost in the parallel agenda of bringing order to the global development 'circus' [30]. As IHP + has realised, the focus of coordination for health, in the long run, is the local: global initiatives need to serve that. But there is a second task of coordination at the global level, and that deals with the complex and unwieldy apparatus of what is now being termed 'development cooperation'.

Severino and Ray redefine this task as steering this global complexity towards greater effectiveness, rather than attempting to reduce or even manage it, as the Paris Declaration has attempted to do. They propose five concurrent modes of collaboration, which may yet come to represent the next adaptation of the coordination agenda:

1. Rules and agreements

2. Norms and standards

3. Systems of incentives

4. Information and discourses

5. Networks and partnerships [38].

Their proposal opens up the global partnership for development. It does not discard the need for regulation and norm-setting, though it does seek a re-imagining of what these might be. It advocates the open and independent evaluation of public policy performance, anticipating that international exposure will itself lead to moderation of inappropriate practice. More importantly, it proposes systems of positive incentives for committing to multi-actor convergence, and the engagement of consumers--civil society, governments and final beneficiaries--in open public debate that feeds back into the iterative loops of development action.

The ideas are gaining some traction. The 4^th ^High Level Forum on Aid Effectiveness, in its final declaration, established an agreed framework for development cooperation that embraces traditional donors, South-South co-operators, BRICS, Arab donors, Civil Society Organisations and private funders [42]. Recognising the need to expand from the constraints of understanding the G20 as the only contributors to aid, the engagement of these previously unrecognised actors redefines the current framing of aid effectiveness into the broader concept 'development cooperation'. The "set of principles", that the Forum was seeking, "founded on solid evidence, to guide the new consensus on development cooperation" [43], are not yet clear. They can only emerge from a progressive re-imaging of the dynamics that underpin global development, grounded in a deep understanding of its evolution. But this may offer yet another radical twist in the conceptualisation of coordination in development assistance.

## Abbreviations

GAVI: GAVI Alliance; Global Fund: Global Fund to Fight AIDS: Tuberculosis and Malaria; IHP+: International Health Partnership and Related Initiatives; JANS: Joint Assessment of National Strategies; MDGs: Millennium Development Goals; OECD: Organisation for Economic Cooperation and Development; PRSPs: Poverty Reduction Strategy Papers; SIPs: Sectoral Investment Programmes; SWAps: Sector Wide Approaches

## Competing interests

The authors declare that they have no competing interests.

## Authors' contributions

PSH and RD conceived the review. SB undertook the original literature search under supervision of PSH, and prepared the first draft. PSH redrafted; the paper was reviewed by RD and JH with additional material. PSH undertook the final draft which all authors have approved. All authors have read and approved the final manuscript.
